# The ATPase activity of Asna1/TRC40 is required for pancreatic progenitor cell survival

**DOI:** 10.1242/dev.154468

**Published:** 2018-01-01

**Authors:** Stefan Norlin, Vishal Parekh, Helena Edlund

**Affiliations:** Umeå Centre for Molecular Medicine, Umeå University, SE-901 87 Umeå, Sweden

**Keywords:** Asna1/TRC40, Pancreatic hypoplasia, Pancreatic progenitor cell, Apoptosis, Impaired differentiation, Integrated stress response, Mouse

## Abstract

Asna1, also known as TRC40, is implicated in the delivery of tail-anchored (TA) proteins into the endoplasmic reticulum (ER), in vesicle-mediated transport, and in chaperoning unfolded proteins during oxidative stress/ATP depletion. Here, we show that *Asna1* inactivation in pancreatic progenitor cells leads to redistribution of the Golgi TA SNARE proteins syntaxin 5 and syntaxin 6, Golgi fragmentation, and accumulation of cytosolic p62^+^ puncta. *Asna1^−/−^* multipotent progenitor cells (MPCs) selectively activate integrated stress response signaling and undergo apoptosis, thereby disrupting endocrine and acinar cell differentiation, resulting in pancreatic agenesis. Rescue experiments implicate the Asna1 ATPase activity and a CXXC di-cysteine motif in ensuring Golgi integrity, syntaxin 5 localization and MPC survival. *Ex vivo* inhibition of retrograde transport reproduces the perturbed Golgi morphology, and syntaxin 5 and syntaxin 6 expression, whereas modulation of p53 activity, using PFT-α and Nutlin-3, prevents or reproduces apoptosis in *Asna1*-deficient and wild-type MPCs, respectively. These findings support a role for the Asna1 ATPase activity in ensuring the survival of pancreatic MPCs, possibly by counteracting p53-mediated apoptosis.

## Abstract (precis)

**Summary:** Conditional inactivation of Asna1/TRC40 in pancreatic progenitor cells results in pancreatic agenesis resulting from pancreatic progenitor cell apoptosis, thus revealing a crucial role for Asna1/TRC40 in pancreatic progenitor cell survival.

## INTRODUCTION

Asna1, also known as TRC40, and its yeast homolog GET3 have been implicated in a variety of cellular processes, including proteasome assembly and degradation of ubiquitylated proteins, growth under oxidative or metal stress, membrane trafficking within the secretory pathway, and insulin secretion (Akahane et al., 2013; Auld et al., 2006; Costanzo et al., 2010; Jonikas et al., 2009; Norlin et al., 2016; Schuldiner et al., 2005; Shen et al., 2003). Many of these phenotypes have been attributed to the ability of Asna1/GET3 to mediate the ATP-dependent delivery of tail-anchored (TA) proteins for post-translational insertion into the ER via the CAML/WRB receptor complex, a pathway that has been delineated in cell-free systems (Favaloro et al., 2008; Johnson et al., 2013; Schuldiner et al., 2008; Stefanovic and Hegde, 2007). The physiological range of TA-protein clients that critically rely on Asna1/GET3 for their membrane insertion may, however, be limited (Norlin et al., 2016; Rivera-Monroy et al., 2016). In addition to its ATPase-dependent functions, studies in yeast have suggested an additional role for Asna1/GET3 as a holdase chaperone under conditions of oxidative stress and ATP depletion (Powis et al., 2013; Voth et al., 2014). The shift from an ATPase-dependent function to the holdase function is associated with a structural reorganization of Asna1/GET3 from a dimer to a tetramer, and involves the oxidation of two di-cysteine motifs (Voth et al., 2014). In *Get3* yeast mutants, the holdase function can be selectively rescued by *Asna1/Get3* constructs that carry mutations in the ATPase domain or hydrophobic groove, i.e. domains that mediate TA-protein insertion (Voth et al., 2014), suggesting that the part of Asna1 that ensures the holdase function is distinct from that required for the ATPase-dependent and TA-targeting activities. In *asna-1-*deficient *C. elegans*, re-expression of *asna-1* mutated in the CXXC di-cysteine motif rescues the severe growth phenotype displayed by worms lacking *asna-1* but not the sensitivity to cisplatin, an oxidative stress-inducing drug (Hemmingsson et al., 2010), suggesting that Asna1 also performs multiple functions in higher eukaryotes. The relative contribution of these functions in mammalian cells *in vivo* remains, however, unknown.

Through conditional inactivation of *Asna1* in insulin-producing β-cells of mice, we recently demonstrated a role for *Asna1* in ensuring retrograde transport and thereby ER homeostasis and insulin biosynthesis in β-cells (Norlin et al., 2016). Notably, the proposed Asna1 target TA proteins syntaxin 5 (Stx5) and syntaxin 6 (Stx6) were redistributed from their Golgi compartments both in *Asna1* mutant β-cells and after pharmacological inhibition of retrograde transport using Retro-2 (Norlin et al., 2016; Stechmann et al., 2010). Together, these findings suggested a key role for *Asna1* in ensuring retrograde transport and Golgi localization of Stx5 and Stx6 in adult β-cells. To gain further insight into the role(s) for *Asna1* in mammalian cells *in vivo*, we have here conditionally inactivated *Asna1* in pancreatic progenitor cells.

Pancreatic development is initiated as two evaginations from the primitive gut epithelia. Over time, the specified pancreatic epithelia grow into the surrounding mesenchyme and form a tubular epithelium that undergoes extensive branching morphogenesis. Mouse pancreatic progenitor cells undergo two major rounds of differentiation (Shih et al., 2013). During the early phase between E9-12 (i.e. 1st transition), multipotent progenitor cells (MPCs), which are capable of generating acinar, endocrine and ductal cell lineages, proliferate and generate a small number of endocrine cells primarily expressing glucagon. During the 2nd transition between E12-14, pancreatic progenitor cells undergo extensive growth and branching morphogenesis, and the initial Ptf1a^+^/Sox9^+^ MPC population segregates into two populations: a branch tip population containing Ptf1a^High^/Sox9^Low^ proacinar cells; and a bipotential Ptf1a^−^/Sox9^High^ branch trunk population containing Ngn3^+^ proendocrine cells and Ngn3^−^ duct progenitor cells (Schaffer et al., 2010; Solar et al., 2009). After E14.5, Ptf1a^High^ proacinar tip cells differentiate and initiate expression of mature acinar cell markers, e.g. amylase. In the branch trunks, duct progenitor cells form the pancreatic ducts that connect the acinar cells to the intestine, whereas the Ngn3^+^ proendocrine cells migrate into the surrounding mesenchyme and initiate expression of endocrine hormones as they differentiate into α-, β-, δ-, γ- and ε-cells that eventually form the endocrine islets. Thus, the different cell types in the developing pancreas serves as a model for evaluating the requirement for *Asna1* in several cellular processes, including proliferation, differentiation, morphogenesis and hormone secretion.

Here, we show that inactivation of *Asna1* in pancreatic progenitor cells results in severe pancreatic agenesis. Loss of *Asna1* in pancreatic progenitor cells leads to rapid redistribution of the TA proteins Stx5 and Stx6, followed by perturbed Golgi morphology, apoptotic cell death, and impaired acinar and endocrine cell differentiation from E13 onwards. In contrast, *Asna1*-deficient Sox9^+^ duct/MPC-like cells, as well as differentiated α-cells and β-cells specified at earlier stages of pancreatic development, survive. Apoptotic cell death in embryonic *Asna1*-deficient pancreatic epithelium correlates with an increase in integrated stress response (ISR) signaling, whereas the adaptive UPR and oxidative stress responsive pathways appear unaltered. Pharmacological inhibition of retrograde transport reproduces the redistribution of Stx5 and Stx6, and the fragmentation of the Golgi, but is insufficient to provoke substantial apoptosis and impaired acinar and endocrine cell differentiation. Re-introduction of an ATPase-deficient mutant of *Asna1* failed to restore Golgi integrity and differentiation of pancreatic progenitor cells lacking *Asna1*, suggesting that the ATPase-dependent and TA-targeting activities of Asna1 are required for pancreatic progenitor cell survival.

## RESULTS

### Deletion of *Asna1* in pancreatic progenitor cells leads to severe pancreatic hypoplasia due to apoptosis

**Fig. 1.**
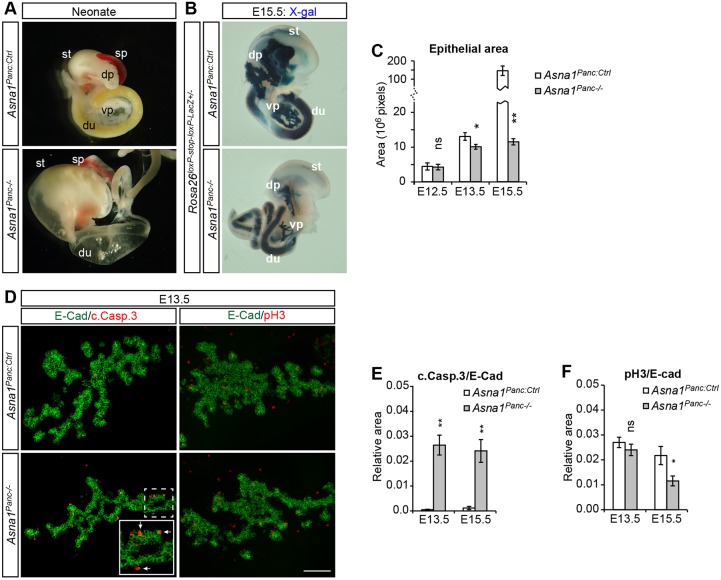
***Asna1^Panc−/−^* mice develop pancreatic and duodenal agenesis due to apoptosis.** (A) Upper gastrointestinal tract dissected from neonatal *Asna1^Panc:Ctrl^* and *Asna1^Panc−/−^* littermates showing pancreatic and duodenal agenesis. (B) X-gal staining of E15.5 *Asna1^Panc:Ctrl^* and *Asna1^Panc:−/−^* embryos on a *Rosa26^loxP−stop-loxP-LacZ^* background. (C) Quantification of the dorsal pancreatic epithelia (E-cad^+^) area of *Asna1^Panc−/−^* and control littermates at E12.5 (*n*=5 and 7, respectively), E13.5 (*n*=6 and 7, respectively) and E15.5 (*n*=5). (D) Representative immunohistochemistry of dorsal pancreatic sections from E13.5 *Asna1^Panc:Ctrl^* and *Asna1^Panc−/−^* embryos using antibodies against E-cadherin (E-cad, green), cleaved caspase 3 (c.Casp.3, red) and phospho-Histone H3 (pH3, red). Inset shows magnification of *Asna1^Panc−/−^* epithelia with cleaved caspase 3^+^ cells at epithelial protrusions (arrows). Scale bar: 100 µm in D. (E) Quantification of cleaved caspase 3^+^ cells relative to E-cad^+^ epithelial area in the dorsal pancreatic epithelia of *Asna1^Panc−/−^* and control littermates at E13.5 (*n*=5 and 7, respectively) and E15.5 (*n*=5). (F) Quantification of phospho-histone H3^+^ (pH3^+^) cells relative to E-cad^+^ epithelial area in the dorsal pancreatic epithelia of *Asna1^Panc−/−^* and control littermates at E13.5 (*n*=5 and 7, respectively) and E15.5 (*n*=5). dp, dorsal pancreas; du, duodenum; vp, ventral pancreas; sp, spleen; st, stomach. Data are presented as mean±s.e.m.; *P<0.05, **P<0.01, ns, not significant (Student's *t*-test).

*Asna1* was broadly expressed in the developing pancreatic epithelium from ∼E10.5 and by E13.5 the expression became prominent in the pro-acinar branch tip cells (Fig. S1A). To elucidate a potential functional role of *Asna1* in mouse pancreatic progenitor cells *in vivo*, we crossed *Asna1^flox/flox^* mice (Norlin et al., 2016) with *Ipf1-nlsCre* mice (Svensson et al., 2009), yielding *Asna1^flox/flox^;Ipf1-nlsCre^+^* mice (denoted *Asna1^Panc−/−^*) (Fig. S1B). *Ipf1-nlsCre* ensures pancreas- and duodenal-specific Cre-mediated recombination in *Rosa26^loxP-stop-loxPLacZ^* reporter mice (Soriano, 1999) as early as E10.5 (Fig. S1C) and, in agreement with this, qRT PCR analysis revealed 68% reduction of *Asna1* expression in pancreatic epithelium of *Asna1^Panc−/−^* embryos at E11.5 (Fig. S1D). *Asna1^Panc WT^* and *Asna1^Panc+/−^* embryos did not show any apparent phenotype at any stage examined and were thus used as controls, collectively denoted *Asna1^Panc:Ctrl^*. The resulting *Asna1^Panc−/−^* mice were born alive but died soon after birth due to severe pancreatic and duodenal agenesis (Fig. 1A).

To identify the cause of the pancreatic agenesis in *Asna1^Panc−/−^* embryos, we next characterized the developing dorsal pancreatic epithelia (DPE) in more detail. X-gal staining of *Asna1^Panc−/−^;Rosa26^loxP-stop-loxPLacZ^* embryos revealed that severe dorsal and ventral pancreatic agenesis and reduced branching complexity were already evident by E15.5 (Fig. 1B). Analyses of earlier developmental stages showed that the size of *Asna1^Panc−/−^* DPE was normal at E12.5, but reduced by 28% at E13.5 (Fig. 1C). Moreover, the rapid expansion of the pancreatic epithelia area of control mice between E13.5 and E15.5 was severely impaired in the *Asna1^Panc−/−^* DPE (Fig. 1C). Notably, the growth defect of *Asna1^Panc−/−^* DPE coincided with the prominent appearance of apoptotic cells at E13.5, preferentially in branch tips along the epithelial/mesenchymal border of the *Asna1^Panc−/−^* DPE (cleaved caspase 3^+^ cells in Fig. 1D,E and TUNEL^+^ cells in Fig. S2A). In contrast, proliferation, as judged by the amount of phospho-H3 (pH3)-positive cells in the *Asna1^Panc−/−^* DPE, was unaffected at E13.5. (Fig. 1F). At E15.5, apoptosis was still drastically increased in *Asna1^Panc−/−^* DPE when compared with *Asna1^Panc:Ctrl^* DPE and, in addition, proliferation was reduced in *Asna1^Panc−/−^* DPE at this stage (Fig. 1E,F). Apoptosis was also evident in E13.5 *Asna1^Panc−/−^* ventral pancreatic and E14.5 *Asna1^Panc−/−^* duodenal epithelia (Fig. S2B). These data show that *Asna1^Panc−/−^* mice develop pancreatic and duodenal epithelial hypoplasia as a consequence of apoptosis.

### Loss of *Asna1* impairs pancreatic acinar, endocrine and ductal cell lineages

**Fig. 2.**
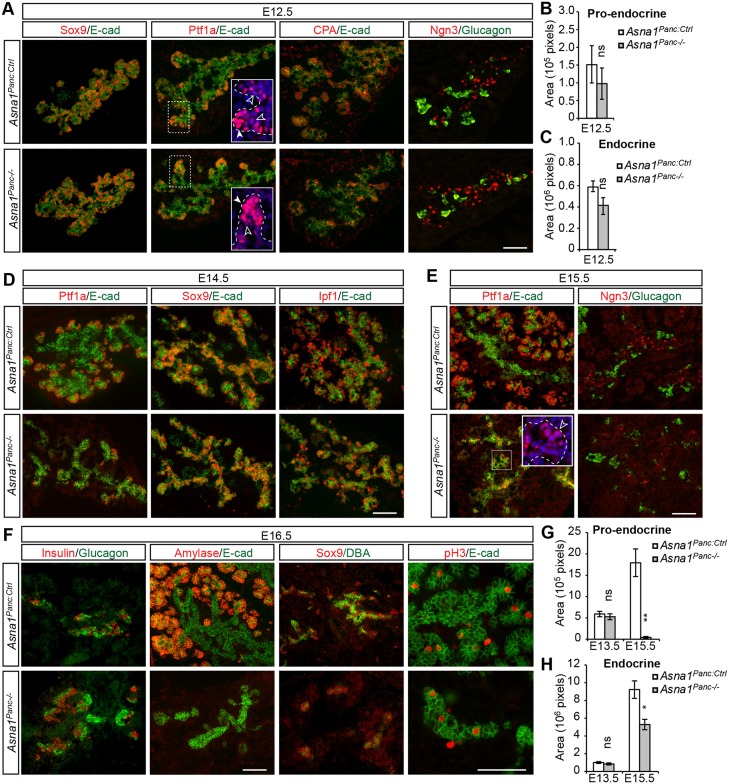
**Impaired endocrine and acinar differentiation in *Asna1^Panc−/−^* DPE.** (A) Immunohistochemistry of dorsal pancreatic sections from E12.5 *Asna1^Panc:Ctrl^* and *Asna1^Panc−/−^* embryos (*n*=3) using antibodies against Sox9, Ptf1a, CPA and Ngn3 (all red), and E-cadherin (E-cad) and glucagon (both green). Insets show Ptf1a^High^ acinar progenitors (white arrowheads) and Ptf1a^Low^ putative MPCs (black arrowheads). (B,C) Quantification of total pro-endocrine (Ngn3^+^) area (B) and total endocrine (glucagon^+^) area (C) in the DPE from *Asna1^Panc:Ctrl^* and *Asna1^Panc−/−^* embryos at E12.5 (*n*=3). (D-F) Immunohistochemistry of dorsal pancreatic sections from E14.5 (D), E15.5 (E) and E16.5 (F) *Asna1^Panc:Ctrl^* and *Asna1^Panc−/−^* embryos (*n*=3) using antibodies against Ptf1a, Sox9, Ipf1, Ngn3, insulin, amylase and phospho-histone H3 (pH3) (all red), and E-cadherin (E-cad) and glucagon or DBA lectin (DBA) (all green). Inset in E shows Ptf1a^Low^ cells (black arrowhead). (G,H) Quantification of total pro-endocrine (Ngn3^+^) area (G) and total endocrine (glucagon^+^+insulin^+^) area (H) in the DPE from *Asna1^Panc:Ctrl^* and *Asna1^Panc−/−^* embryos at E13.5 and E15.5 (*n*=5, respectively). Data are mean±s.e.m., *P<0.05, **P<0.01, ns, not significant (Student's *t*-test). Scale bars: 100 µm in A,D-F

To identify the pancreatic cell types that undergo apoptosis in *Asna1^Panc−/−^* DPE, we next analyzed the expression of cell specification and differentiation markers in E12.5-E16.5 *Asna1^Panc−/−^* DPE. Cells in the branch trunk regions of E12.5 *Asna1^Panc−/−^* DPE showed normal Sox9 expression that is characteristic of endocrine and ductal progenitor cells (Fig. 2A). Moreover, like that of control littermates, branch tips of *Asna1^Panc−/−^* DPE contained a subset of cells that showed Ptf1a^High^ expression characteristic of pro-acinar cells, and Ptf1a^Low^ and carboxypeptidase A^+^ (CPA^+^) cells that may represent Ptf1a^Low^/Sox9^+^/CPA^+^ MPCs (Pan et al., 2013; Zhou et al., 2007) (Fig. 2A). Ngn3^+^ endocrine progenitor cells and differentiated glucagon^+^ α-cells were dispersed within and around the branch trunk region (Fig. 2A), and quantification revealed no significant difference in the numbers of these cells at E12.5, i.e. prior to the onset of apoptosis, when comparing *Asna1^Panc−/−^* and control littermates (Fig. 2B,C). Thus, the initial lineage specification of MPCs, endocrine and ductal progenitor cells, as well as differentiation of α-cells is unaffected in *Asna1^Panc−/−^* mice up to E12.5.

Following the onset of apoptosis at E13.5, branching morphogenesis was clearly perturbed by E14.5 onwards (Fig. 2D-F). The expression of Sox9 and Ipf1/Pdx1 appeared unaltered in the bipotential branch trunk progenitors, arguing against a role for these transcription factors in mediating impaired morphogenesis and progenitor cell apoptosis in E14.5 *Asna1^Panc−/−^* DPE (Fig. 2D). Consistent with the localization of apoptotic cells in branch tips of the E13.5 DPE (Fig. 1D, Fig. S2A), Ptf1a^+^ tip cells appeared reduced in number in *Asna1^Panc−/−^* DPE at E14.5 (Fig. 2D). Moreover, although some Ptf1a^Low^ cells remained at E15.5 (Fig. 2E insets), Ptf1a^High^ pro-acinar cells were essentially absent (Fig. 2E) and the number of pro-endocrine Ngn3^+^ cells was severely reduced at this stage (Fig. 2E,G). Together, these results provide evidence that *Asna1* is required for survival of MPCs and consequently their progression into Ptf1a^High^ pro-acinar and Ngn3^+^ pro-endocrine cells. Coherent with this scenario, definitive amylase^+^ acinar cells were essentially absent, and numbers of differentiated glucagon^+^ α-cells and insulin^+^ β-cells were significantly reduced in E15.5-E16.5 *Asna1^Panc−/−^* DPE (Fig. 2F,H, Fig. S3). Notably, the number of α-cells and β-cells still increased in *Asna1^Panc−/−^* DPE between E13.5 and E15.5 (Fig. 2H), suggesting that they derive from Ngn3^+^ pro-endocrine cells already specified at E13.5. The remaining E16.5 *Asna1^Panc−/−^* epithelium consisted of proliferating Sox9^Low^/DBA-lectin^Low^ cells (Fig. 2F), which survive and differentiate in the absence of *Asna1*. Taken together, these data suggest that MPCs and/or their immediate offspring undergo apoptosis from E13.5 onwards in *Asna1^Panc−/−^* embryos, which impairs the generation of pro-endocrine and pro-acinar cells, and, consequently, the subsequent differentiation of endocrine and acinar cell types.

### *Asna1^Panc−/−^* pancreatic agenesis is associated with p53 activity and integrated stress response signaling

**Fig. 3.**
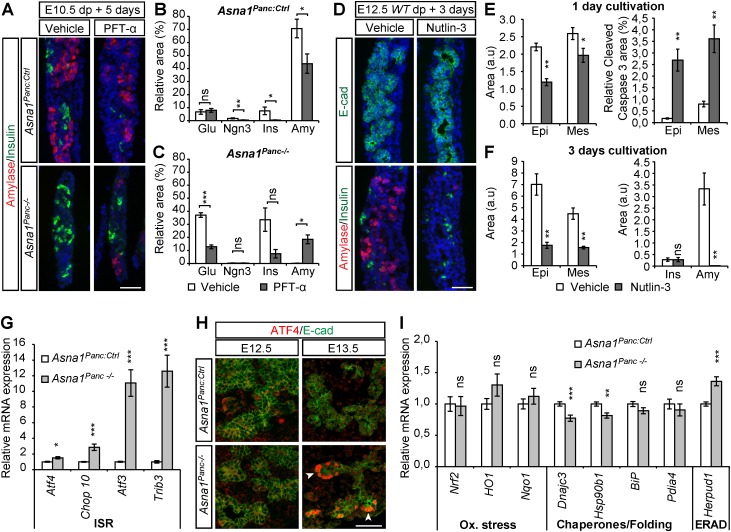
**Apoptosis of pro-acinar progenitors in *Asna1^Panc−/−^* DPE is associated with p53 activity and activation of the IRS.** (A) Representative immunohistochemistry of dorsal pancreas explants from E10.5 *Asna1^Panc:Ctrl^* and *Asna1^Panc−/−^* cultivated for 5 days exposed to vehicle (*n*=7 and 4, respectively) or PFT-α (30 µM) (*n*=11 and 4, respectively) using antibodies against amylase (red) and insulin (green). (B,C) Quantification of experiments described in A. Relative glucagon^+^ (Glu), Ngn3^+^, insulin^+^ (Ins) and amylase^+^ (Amy) areas over total E-cadherin^+^ area in explants from *Asna1^Panc:Ctrl^* (B) and *Asna1^Panc−/−^* (C) mice. (D) Representative immunohistochemistry of dorsal pancreas explants from E12.5 wild-type embryos cultivated for 3 days exposed to vehicle or Nutlin-3 (10 µM), using antibodies against E-cadherin (E-cad) and insulin (both green), and amylase (red). (E,F) Quantification of experiments described in D after 1 day (*n*=6 for each condition) (E) and 3 days (vehicle, *n*=4; Nutlin-3, *n*=5) (F) of cultivation; E-cadherin^+^ epithelial (Epi) and E-cadherin^−^ mesenchymal (Mes) area; relative cleaved caspase 3^+^ (c.Casp.3) over E-cadherin^+^ area after 1 day; absolute insulin^+^ (Ins) and amylase^+^ (Amy) area after 3 days; arbitrary units (a.u). (G) qRT-PCR mRNA levels of the indicated ISR genes in dorsal pancreatic buds from E13.5 *Asna1^Panc:Ctrl^* (*n*=7) and *Asna1^Panc−/−^* (*n*=6) embryos. (H) Immunohistochemistry of dorsal pancreatic sections from E12.5 and E13.5 *Asna1^Panc:Ctrl^* and *Asna1^Panc−/−^* embryos (*n*=5) using antibodies against ATF4 (red) and E-cadherin (E-cad, green). Arrowheads indicate ATF4^+^ tip cells. (I) qRT-PCR mRNA levels of the indicated UPR genes in dorsal pancreatic buds from E13.5 *Asna1^Panc:Ctrl^* and *Asna1^Panc−/−^* (*n*=7) and *Asna1^Panc−/−^* (*n*=6) embryos. DAPI (blue) indicates nuclei in A,C. Scale bars: 50µm. Data are mean±s.e.m.; *P<0.05, **P<0.01, ***P<0.001; ns, not significant (Student's *t*-test).

Mouse genetic models presenting with severe pancreatic agenesis and acinar hypoplasia appear to be preferentially associated with premature differentiation, impaired proliferation or altered specification (Apelqvist et al., 1999; De Vas et al., 2015; Fukuda et al., 2008; Jensen et al., 2000; Krapp et al., 1996; Murtaugh et al., 2005; Nakhai et al., 2008; Papadopoulou and Edlund, 2005; Seymour et al., 2007) and not primarily with apoptosis. However, pancreas-specific ablation of DNA methyltransferase 1 (Dnmt1) (Georgia et al., 2013) was shown to provoke pancreatic progenitor cell apoptosis, which involved the de-repression of *p53*, an oncogene that induces cell cycle block or apoptosis in response to cellular stress such as genotoxic and ER stress (Brooks and Gu, 2010; Vogelstein et al., 2000). To investigate a potential role for p53-mediated apoptosis in *Asna1^Panc−/−^* DPE, we modulated p53 activity in explants of wild-type and *Asna1^Panc−/−^* pancreas. Exposure of E10.5 *Asna1^Panc:Ctrl^* explants to the p53 inhibitor pifithrin-α (PFT-α) (Komarov et al., 1999) resulted in a reduced Ngn3^+^, insulin^+^ and amylase^+^ cell area, implying a requirement for p53 in β-cell and acinar cell differentiation (Fig. 3A,B). Similarly, β-cell differentiation was reduced in *Asna1^Panc−/−^* explants exposed to PFT-α (Fig. 3A,C). In addition, glucagon^+^ area was also reduced, suggesting that p53 may play a role in α-cell survival and/or differentiation in *Asna1^Panc−/−^* embryos. In contrast, however, amylase^+^ cell differentiation was, albeit partially, restored in *Asna1^Panc−/−^* explants exposed to PFT-α (Fig. 3A,C), suggesting that excess p53 activation may provoke the apoptosis of *Asna1^Panc−/−^* MPCs. To test this idea, we exposed wild-type E12.5 explants to the p53 agonist Nutlin-3 (Vassilev et al., 2004) (Fig. 3D), which resulted in increased epithelial apoptosis after 24 h (Fig. 3E) and a dramatic reduction of epithelial area and in amylase^+^ cell number after 3 days (i.e. E15.5) (Fig. 3D,F). Insulin^+^ cells appeared unaffected (Fig. 3F). Total p53 protein levels in E13.5 dorsal pancreas buds, however, were not increased (Fig. S4), suggesting that p53 activity, rather than expression levels, were increased in *Asna1^Panc−/−^* DPE. Taken together, these results leave open the possibility that apoptosis in *Asna1^Panc−/−^* DPE, at least in part, is mediated by p53.

As inactivation of *Asna1* in mouse, *C. elegans* and yeast has been associated with both ER stress and increased sensitivity to oxidative stress, we next investigated stress signaling responses in *Asna1^Panc−/−^* embryonic pancreatic epithelium. Several cellular stress responses converge on the phosphorylation of eIF2α that, together with its downstream effector genes, are referred to collectively as the integrated stress response (ISR) pathway. qRT-PCR analysis of *Asna1^Panc−/−^* dorsal pancreatic buds, i.e. pancreatic epithelia and surrounding mesenchyme, revealed that the expression of genes associated with the ISR, such as *Atf4* and its target genes *Chop10* (*Ddit3* – Mouse Genome Informatics), *Atf3* and *Trib3*, was increased at E13.5 (Fig. 3G). Additionally, immunohistochemical analyses revealed strong nuclear expression of ATF4 in tip cells of *Asna1^Panc−/−^* DPE at E13.5, but not at E12.5 (Fig. 3H), thus correlating with the appearance of apoptotic progenitor cells (Fig. 1D). Taken together, these results suggest that ISR signaling and apoptosis occur in a spatially restricted subset of *Asna1^Panc−/−^* DPE cells.

The expression of oxidative stress genes, e.g. *Nrf2* and its target genes *Nqo1* and *Hmox1* (previously *HO-1*), appeared unaltered at E13.5 (Fig. 3I). Although the expression of the UPR-regulated gene *Herpud1*, which encodes a component of the ER-associated degradation (ERAD) machinery, was upregulated in *Asna1^Panc−/−^* DPE, the expression of genes associated with the adaptive UPR, which encode chaperones [*DnaJc3*, *Hsp90b1* (i.e. *Grp94*) and *BiP* (*Hspa5*) and oxidative folding enzymes (*Pdia4*)] rather showed a tendency to be reduced in E13.5 pancreatic buds (Fig. 3I), arguing against ER stress as the underlying cause for apoptosis observed in *Asna1^Panc−/−^* DPE. Together, these data suggest that in *Asna1^Panc−/−^* DPE tip cells, the ISR activates a program, possibly involving p53, that leads to MPC apoptosis and subsequent pancreatic agenesis.

### Redistribution of Stx5 and Stx6 precedes Golgi fragmentation and apoptosis in *Asna1^Panc−/−^* pancreatic and duodenal epithelia

**Fig. 4.**
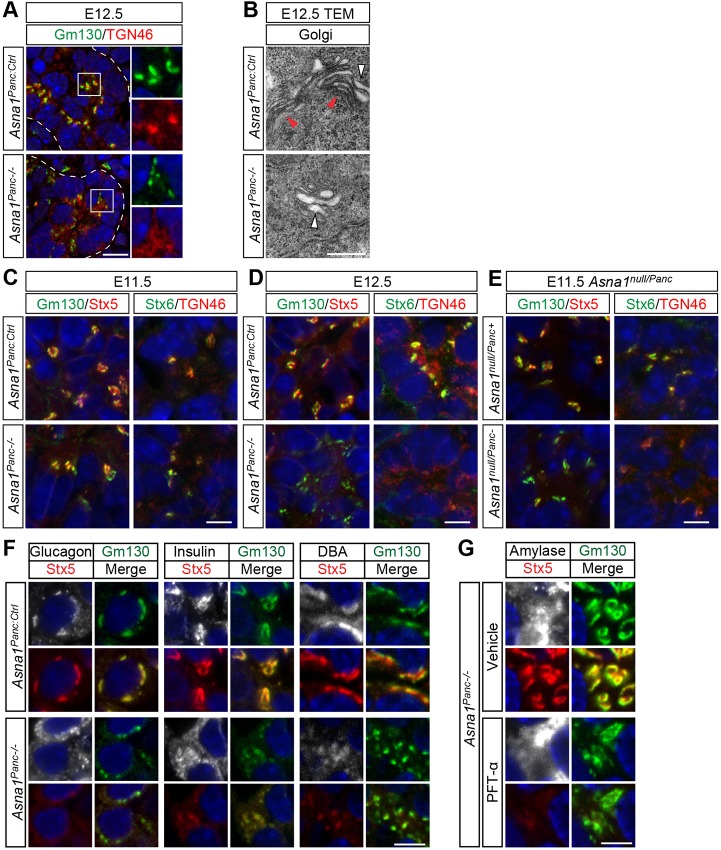
**Altered morphology and Stx5 and Stx6 expression in the Golgi apparatus of *Asna1^Panc−/−^* DPE cells.** (A) Immunohistochemistry of dorsal pancreatic sections from E12.5 *Asna1^Panc:Ctrl^* and *Asna1^Panc−/−^* embryos (*n*=3) using antibodies against Gm130 (green) and TGN46 (red). Boxed areas are shown at higher magnification on the right. (B) Transmission electron micrograph (TEM) of dorsal pancreatic sections from E12.5 *Asna1^Panc:Ctrl^* and *Asna1^Panc−/−^* embryos (*n*=3) showing compact Golgi stacks (red arrowheads) and distended Golgi stacks (white arrowheads). (C-E) Immunohistochemistry of dorsal pancreatic sections from E11.5 (C,E) or E12.5 (D) *Asna1^Panc:Ctrl^* and *Asna1^Panc−/−^* embryos (*n*=3) (C,D) or *Asna1^null/Panc+^* and *Asna1^null/Panc−^* (E) embryos (*n*=3), using antibodies against Gm130 (green), Stx5 (red), Stx6 (green) and TGN46 (red). (F) Immunohistochemistry of dorsal pancreatic sections from E16.5 *Asna1^Panc:Ctrl^* and *Asna1^Panc−/−^* embryos (*n*=3) using antibodies against Stx5 (red) and Gm130 (green) together with either glucagon, insulin or DBA-lectin (all white). Merge panels show co-expression between Stx5 and Gm130 (yellow). (G) Immunohistochemistry of dorsal pancreas explants from E10.5 *Asna1^Panc−/−^* cultivated for 5 days (*n*=3) exposed to vehicle or PFT-α, using antibodies against Stx5 (red) and Gm130 (green) together with amylase (white). Merge panels show co-expression between Stx5 and Gm130 (yellow). DAPI (blue) indicates nuclei in A,C-G. Scale bars: 50 µm in A; 0.5 µm in B; 10 µm in C-E; 5 µm in F,G.

We next tried to establish a connection between the previously proposed functions of Asna1, ISR signaling and pancreatic progenitor cell apoptosis. As Asna1 is implicated in the targeting of TA proteins to the ER membrane, we first analyzed the structural integrity of endomembrane. Because apoptosis itself is associated with the breakdown of cellular structures, we analyzed the structural integrity of endomembrane compartments in *Asna1^Panc−/−^* DPE at E12.5, i.e. 1 day prior to the onset of apoptosis. Immunohistochemical analyses using markers for the ER (KDEL), endoplasmic reticulum Golgi-intermediate compartment (ERGIC53), endosome (EEA1) and lysosome (Lamp1) revealed that these compartments appeared structurally indistinguishable from that of controls (Fig. S5A). However, analyses using markers for the cis-Golgi (Gm130) and trans-Golgi network (TGN) (TGN46) showed that these markers were uncharacteristically dispersed in the cytoplasm of cells throughout the *Asna1^Panc−/−^* DPE (Fig. 4A). Transmission electron microscopy (TEM) revealed the presence of small distended membrane stacks in *Asna1^Panc−/−^* DPE, as opposed to the thin multilayered membrane stacks characteristic of the cis- and medial Golgi compartment observed in control littermates (Fig. 4B). However, no apparent difference was observed in ER or mitochondrial morphology at E12.5 (Fig. S5B). Together, these results show that cis-Golgi and TGN morphology is perturbed in cells throughout the *Asna1^Panc−/−^* DPE prior to apoptosis.

Loss of Asna1/GET3 function in both yeast and mouse is associated with altered subcellular localization of the TA-SNAREs Stx5/Sed5 and Stx6, which undergo a striking re-localization from the cis-Golgi and TGN, respectively (Jonikas et al., 2009; Norlin et al., 2016; Schuldiner et al., 2008). In *Asna1^Panc−/−^* DPE at E11.5, both Golgi integrity, as well as Stx5 and Stx6 expression, was normal (Fig. 4C). However, at E12.5, Stx5 and Stx6 immunoreactivity was severely reduced and virtually absent in the fragmented cis-Golgi and TGN compartments of *Asna1^Panc−/−^* DPE (Fig. 4D). However, total Stx5 protein levels were unaltered in E12.5 pancreas buds (Fig. S5C), suggesting that, like Asna1-deficient β-cells (Norlin et al., 2016), Stx5 protein becomes redistributed rather than downregulated in *Asna1^Panc−/−^* DPE. An intermediate phenotype was observed in embryos carrying a germline *Asna1^null^* allele in combination with a conditionally inactivated *Asna1^flox^* allele, denoted *Asna1^null/Panc−^*, in which Stx5 and Stx6 immunoreactivity was already perturbed at E11.5, whereas Golgi compartments appeared intact (Fig. 4E). Nonetheless, this earlier depletion of Asna1 in *Asna1^null/Panc−^* embryos was insufficient to provoke an earlier onset of increased apoptosis at E12.5 (Fig. S5D). Taken together, these results provide evidence that redistribution of Stx5 and Stx6 precedes Golgi fragmentation, which in turn is followed by overt apoptosis at E13.5. Moreover, similar changes in Golgi morphology and syntaxin distribution also preceded apoptosis in the ventral pancreas and the duodenum epithelia (Fig. S5E,F), suggesting a general *Asna1*-dependent function(s) in endodermal progenitor cells.

We next investigated whether Stx5 redistribution and Golgi fragmentation was retained in surviving cell types at E16.5. The Sox9^Low^/DBA-lectin^Low^ duct-like cells, β-cells and most α-cells all showed redistribution of Stx5 and fragmented Golgi compartments (Fig. 4F), suggesting that Stx5 redistribution and Golgi fragmentation does not affect survival of these cells. Notably, as the *Ipf1-nlsCre* transgene is inactive in α-cells, these results further suggest that most α-cells are derived from *Asna1*-deficient Ngn3^+^ pro-endocrine cells. However, a minor fraction of the α-cells showed intact Golgi structure and Stx5 localization (data not shown), suggesting that they derive from the early pool of α-cells that are generated prior to E11.5, and may thus have escaped *Ipf1-nlsCre*-mediated inactivation of *Asna1*. Finally, treatment of *Asna1^Panc−/−^* explants with the p53 inhibitor PFT-α did not restore Stx5 distribution in epithelial cells, including the rescued amylase^+^ cells (Fig. 4G), suggesting that p53 inhibition did not prevent apoptosis by restoring Stx5 localization or by interfering with CRE-mediated inactivation of *Asna1*. However, the integrity of the Golgi compartment in the rescued amylase^+^ cells appeared partially restored (Fig. 4G). These results demonstrate that *Asna1* inactivation, Stx5 redistribution and Golgi fragmentation is tolerated by most cell types in the developing pancreas, thus highlighting the selective sensitivity of MPCs.

### p62^+^ protein aggregates accumulate in *Asna1^Panc−/−^* DPE

**Fig. 5.**
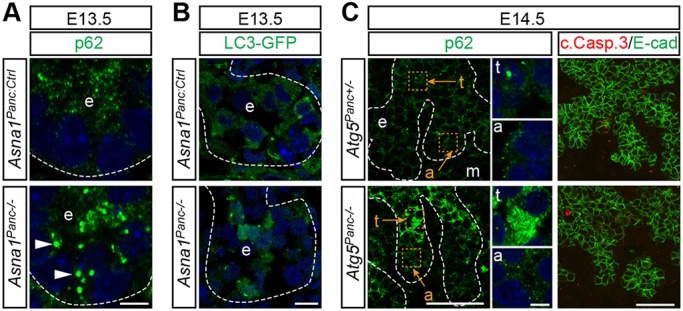
**Accumulation of autophagy markers in *Asna1^Panc−/−^* DPE.** (A) Immunohistochemistry of dorsal pancreatic sections from E13.5 *Asna1^Panc:Ctrl^* and *Asna1^Panc−/−^* embryos (*n*=5) using antibodies against p62 (green). Arrowheads indicate p62^+^ puncta. (B) Dorsal pancreatic sections from E13.5 *Asna1^Panc:Ctrl^* and *Asna1^Panc−/−^* embryos on a *LC3-GFP* transgenic background, showing LC3-GFP fluorescence in green (*n*=2). (C) Immunohistochemistry of dorsal pancreatic sections from E14.5 *Atg5^Panc+/−^* and *Atg5^Panc−/−^* embryos (*n*=3), using antibodies against p62 (green), E-cadherin (E-cad; green) and cleaved caspase3 (c.Casp.3; red). Insets show magnification of selected trunk (t) and acinar (a) epithelia areas (orange squares, letters and arrows). DAPI (blue) indicates nuclei in A-C. Dashed lines delineate pancreatic epithelium (e) and mesenchyme (m). Scale bars: 5 µm in A,B and insets in C; 50 µm in C. All image pairs were captured using the same settings.

Apart from a role in TA-protein targeting, endomembrane transport and ER homeostasis, Asna1/GET3 is also implicated as a redox-regulated holdase chaperone during oxidative stress and ATP depletion (Powis et al., 2013; Voth et al., 2014). Thus, one consequence of *Asna1* inactivation might be the aggregation of misfolded TA, as has been suggested for Stx5 (Rivera-Monroy et al., 2016), and/or non-TA Asna1 target proteins, which may be potentially cytotoxic. In keeping with this idea, the E13.5 pancreatic epithelium showed increased numbers of distinct puncta positive for p62 (Fig. 5A), an ubiquitin-like protein conjugate that targets proteins for autophagic degradation. These results suggest that protein inclusions destined for proteasomal and/or autophagic degradation accumulates in *Asna1^Panc−/−^* DPE at the time of apoptotic onset. An alternative explanation could be that autophagic degradation per se is impaired in *Asna1^Panc−/−^* DPE as autophagy is dependent on vesicular transport. To separate these possibilities, we first investigated whether autophagic degradation was decreased by analyzing the accumulation of LC3^+^ autophagosomes in GFP-LC3 reporter mice on an *Asna1^Panc−/−^* background. Although occasional cells exhibited increased GFP-LC3 levels, a general accumulation in LC3-GFP levels was not observed in *Asna1^Panc−/−^* DPE (Fig. 5B). Next, we specifically inactivated autophagy in the developing pancreas by generating *Atg5^flox/flox^*, *Ipf1-nlsCre* mice (denoted *Atg5^Panc−/−^*). At E14.5, *Atg5^Panc−/−^* DPE exhibited an increase in cytosolic p62 protein in the pancreatic trunk epithelia but not in pro-acinar tip cells (Fig. 5C, insets). Notably, no corresponding increase in p62^+^ puncta or apoptosis was observed in any part of the *Atg5^Panc−/−^* DPE (Fig. 5C). Taken together, these results strongly argue against decreased autophagic degradation as the primary cause for the accumulation of p62^+^ puncta in *Asna1^Panc−/−^* cells. Instead, it appears likely that these p62^+^ puncta consist of aggregating Asna1 client TA- or non-TA-proteins, which in turn may contribute to apoptotic cell death.

### Progenitor cell survival and exocrine cell differentiation depend on the ATPase activity of Asna1

**Fig. 6.**
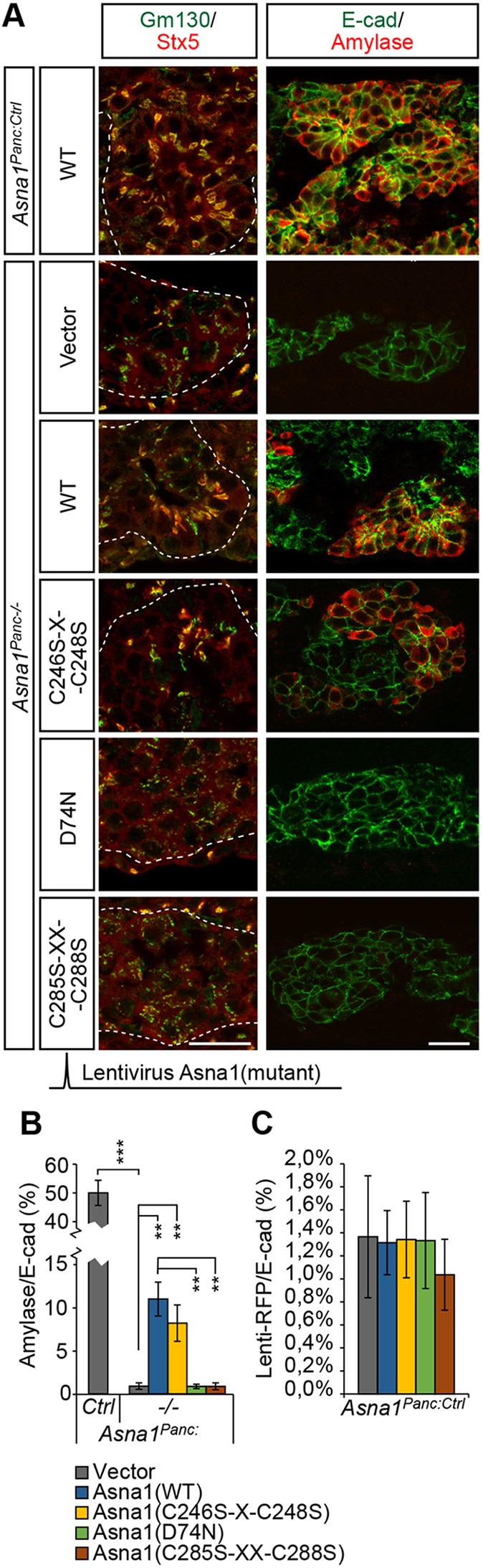
**Normal Golgi morphology, Stx5 distribution and acinar differentiation requires the ATPase domain and CXXC(285-288) di-cysteine motif of Asna1.** (A) Representative immunohistochemistry of lentivirus-infected dorsal pancreas explants using antibodies against Gm130 (green), Stx5 (red), E-cadherin (E-cad; green) and amylase (red). Dorsal pancreas explants from E11.5 *Asna1^Panc:Ctrl^* or *Asna1^Panc−/−^* embryos were transduced with lentivirus particles carrying either empty expression vector (Vector) (*n*=6 and 5, respectively) or constructs encoding wild-type Asna1 (WT) (*n*=5), Asna1 (D74N) (*n*=4), Asna1 (C246S-X-C248S) (*n*=13) or Asna1 (C285S-XX-C288S) (*n*=4), as indicated, and grown for 5 days. Dashed lines delineate pancreatic epithelium. Scale bar: 25 µm. (B) Quantification of relative amylase^+^ epithelial area (amylase^+^/total E-cad^+^) from experiments described in A. (C) Quantification of relative RFP^+^ epithelial area (RFP^+^/Ecad^+^) in control explants infected with the different lentivirus expression constructs from experiments described in A. Data are presented as mean±s.e.m., **P<0.01, ***P<0.001 (Student's *t*-test).

Rescue experiments in yeast have demonstrated that both the ATPase activity and the TA-binding ability are required for the TA-targeting function of GET3/Asna1, as defined by the Golgi localization of Sed5/Stx5, but not for growth under oxidative stress (Voth et al., 2014). The GET3/Asna1 holdase activity is associated with a tetramer configuration and involves the formation of di-sulfide bonds within two di-cysteine motifs, CXC(246-248) and CXXC(285-288) (Voth et al., 2014). Accordingly, the CXXC motif is required for growth under oxidative stress in yeast (Metz et al., 2006). Moreover, in *C. elegans*, the CXXC motif of Asna1 is required for resistance to the oxidative stress-inducing drug cisplatin, but not for rescuing the growth defect of *Asna1* mutant worms (Hemmingsson et al., 2010). *In vitro*, mutations in the CXXC(285-288) motif affect GET3/Asna1 homodimerization, TA-protein binding and ATPase activity (Mateja et al., 2009; Metz et al., 2006), suggesting a possible dual role for the CXXC(285-288) motif in both configurations of Asna1. The ability of the Asna1 CXXC(285-288) mutant to restore Stx5 localization *in vivo* has, however, not been directly tested. The more N-terminal CXC(246-248) motif is not required for dimer formation *in vitro* or for growth under oxidative stress conditions (Metz et al., 2006). To explore which of the activities of Asna1, i.e. the ATPase-dependent chaperone (TA-targeting) or holdase function, is required for pancreatic progenitor survival and differentiation, we next used a lentivirus delivery system to reintroduce *Asna1* versions carrying mutations in different functional domains into *Asna1^Panc−/−^* DPE. E11.5 *Asna1^Panc−/−^* pancreatic explants infected with empty lentivirus control vector and cultivated for 5 days did not show any evidence of exocrine differentiation, the Golgi compartments were fragmented and Stx5 expression was faint (Fig. 6A), thus reproducing the phenotypes of E16.5 *Asna1^Panc−/−^* DPE.

Infection of *Asna1^Panc−/−^* pancreatic explants with *Asna1* wild-type construct partly restored acinar cell differentiation as well as Stx5 expression in the Golgi (Fig. 6A,B). Similar results were obtained when re-expressing the *Asna1(C246S-X-C248S)* mutant (Fig. 6A,B). In contrast, an *Asna1(D74N)* construct, which carries a mutation in the ATPase domain, failed to restore Golgi morphology, Stx5 expression and acinar cell differentiation (Fig. 6A,B). Similar to the *Asna1(D74N)* construct, re-expression of *Asna1(C285S-XX-C288S)* failed to rescue the *Asna1* deficiency in E11.5 *Asna1^Panc−/−^* pancreatic explants (Fig. 6A,B). These results suggest that, in agreement with its proposed role in the dimerization of Asna1 (Mateja et al., 2009; Metz et al., 2006), the CXXC(285-288) motif also affects the ATPase-dependent activities of the Asna1 dimer, and thus cannot be used to assess the contribution of a putative ATPase-independent holdase function of Asna1 in *Asna1^Panc−/−^* mice. Thus, these data show that the ATPase-dependent chaperone functions of Asna1 are required for ensuring both Stx5 expression/localization, as well as the survival of pancreatic progenitor cells from E13.5 onwards.

### Inhibition of retrograde transport in pancreatic progenitor cells mimics Stx5 redistribution and Golgi fragmentation but does not provoke apoptosis

**Fig. 7.**
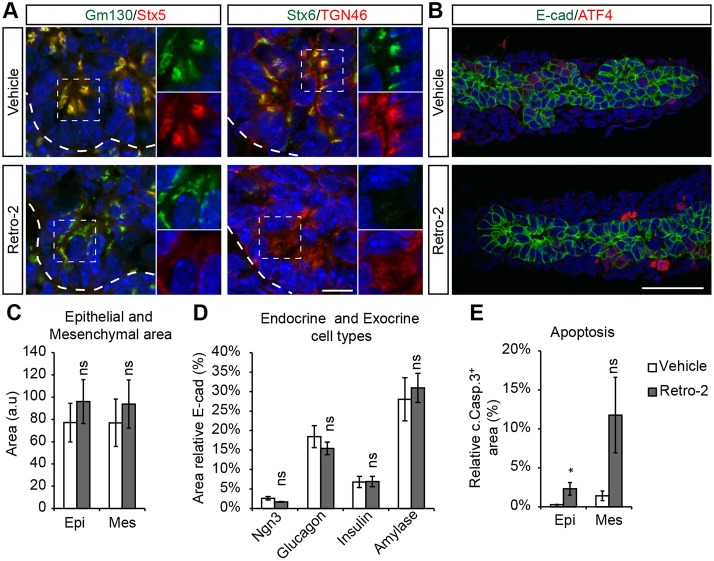
**Inhibition of retrograde transport by Retro-2 disrupts Golgi morphology and Stx5 distribution but allows for acinar differentiation.** (A,B) Immunohistochemistry of E11.5 dorsal pancreas explants from wild-type embryos grown for 48 h and exposed to vehicle (*n*=4) or Retro-2 (50 µM) (*n*=5), using antibodies against Gm130, Stx6 and E-cad (all green), and Stx5, TGN46 and ATF4 (all red). Insets in A show magnification of green and red channels for selected areas (dashed squares). Dashed lines delineate pancreatic epithelium. Scale bars: 10 µm in A; 50 µm in B. (C-E) Quantification of total epithelial (Epi) and mesenchymal (Mes) area (C), relative endocrine (Ngn3^+^, glucagon^+^ and insulin^+^) and exocrine (amylase^+^) area (% of total E-cad^+^ area) (D), and relative apoptotic cell area [% cleaved caspase 3^+^ (c.Casp.3^+^) of E-cad^+^ epithelium (Epi) and E-cad^−^ mesenchyme (Mes)] (E) in E10.5 dorsal pancreas explants grown for 5 days exposed to vehicle (*n*=8) or Retro-2 (50 µM) (*n*=7). Data are presented as mean± s.e.m.; *P<0.05; ns, not significant (Student's *t*-test).

The loss of ATPase/CXXC-dependent activities in *Asna1^Panc−/−^* mice is likely to affect an array of cellular processes, including vesicle-mediated transport of various protein cargo. In yeast, the GET complex, including GET3/Asna1, genetically interacts with retrograde transport pathways (Jonikas et al., 2009; Schuldiner et al., 2005) and β-cell-specific inactivation of *Asna1* result in impaired endosome (EE)-to-TGN as well as COPI-independent Golgi-to-ER transport (Norlin et al., 2016). Thus, we next investigated whether inhibition of retrograde transport with the small molecule inhibitor Retro-2 (Norlin et al., 2016; Stechmann et al., 2010) could reproduce *Asna1^Panc−/−^* phenotypes. Exposure of E11.5 wild-type (wt) dorsal pancreatic bud explants to Retro-2 for 48 h resulted in reduced Stx5 and Stx6 expression in the cis-Golgi and TGN compartments (Fig. 7A). In analogy with *Asna1^Panc−/−^* DPE, the cis-Golgi of Retro-2-treated wild-type DPE appeared partly fragmented and the TGN more diffuse (Fig. 7A, insets). However, Retro-2 exposure did not induce prominent ATF4 expression in DPE explants (Fig. 7B) and extended exposure of E10.5 wild-type DPE explants to Retro-2 for 5 days (i.e. equivalent to E15.5) did not significantly affect epithelial size or the differentiation of endocrine or acinar cell lineages (Fig. 7C,D), although it did provoke apoptosis in both epithelial and mesenchymal cells (Fig. 7E). Taken together, these data show that inhibition of retrograde transport by Retro-2 is sufficient to reproduce the redistribution of Stx5 and Stx6 and the fragmentation of the Golgi compartments but not the loss of acinar and endocrine cell types observed in *Asna1^Panc−/−^* DPE.

## DISCUSSION

Here, we show that inactivation of *Asna1* in pancreatic progenitor cells leads to selective apoptosis of multipotent progenitor cells, thereby depleting the pancreatic progenitor cell pool and perturbing subsequent growth and differentiation of the developing pancreatic epithelium, which ultimately results in severe pancreatic agenesis. Embryonic pancreatic epithelial cells lacking *Asna1* exhibited perturbed localization of Stx5 and Stx6 in the cis-Golgi and TGN compartments, respectively, aberrant Golgi morphology, activation of the ISR and accumulation of cytosolic p62^+^ protein aggregates. Finally, we demonstrate that the ATPase activity of Asna1 is required to restore Golgi morphology and Stx5 expression as well as progenitor cell survival.

The most striking effect of *Asna1* deficiency in pancreatic progenitor cells is the severe pancreatic agenesis that appears to be caused by selective apoptosis of multipotent progenitor cells at E13.5. However, the reduced pancreatic epithelial cell proliferation observed at E15.5, which likely is secondary to the apoptosis of MPC at E13.5, might also contribute to the pancreatic hypoplasia of *Asna1^Panc−/−^* mice. Whereas *Asna1* seems to be functionally depleted throughout the E12.5 *Asna1^Panc−/−^* DPE, as indicated by perturbed Golgi morphology and Stx5 expression, the first signs of apoptosis and increased ISR signaling, i.e. ATF4 expression, is observed 1 day later and is primarily evident in the branch tips of the growing pancreatic epithelium that contain MPCs for both acinar and endocrine cell linages, thus providing an explanation for the perturbed endocrine and acinar cell differentiation in *Asna1^Panc−/−^* pancreatic epithelium beyond E13.5 (Zhou et al., 2007). Moreover, the few remaining endocrine cells observed in *Asna1^Panc−/−^* embryos do not appear to escape recombination as Stx5 and Stx6 are mis-localized also in these cells, providing evidence that loss of *Asna1* per se does not affect survival of already specified lineage-specific progenitor cells nor their subsequent differentiation. Thus, the severe pancreatic hypoplasia observed in *Asna1^Panc−/−^* embryos appears to be the consequence of massive cell death within the domain of multipotent progenitor cells or their immediate offspring from E13.5 onwards.

The Asna1/GET3 pathway has been implicated in multiple functions in yeast and mammalian cells, including that of (1) an ATPase-dependent chaperone function required for Sed5/Stx5 localization to the Golgi (Schuldiner et al., 2008; Voth et al., 2014); (2) an ATP independent holdase chaperone function that supports growth under conditions of oxidative/metal stress (Powis et al., 2013; Voth et al., 2014); and (3) a proteostatic quality control network ensuring cellular proteostasis (Akahane et al., 2013; Auld et al., 2006). Accordingly, *Asna1^Panc−/−^* pancreatic progenitor cells display reduced Stx5 expression in the Golgi, which may be attributed to the loss of ATPase-dependent chaperone activity of Asna1, and increased prevalence of cytosolic p62^+^ puncta, which may be interpreted as an accumulation of protein aggregates in the absence of the holdase activity of Asna1 and/or due to the proposed role for Asna1 in ensuring cellular proteostasis. Notably, our results clearly demonstrate that the ATPase activity of Asna1 is required for restoring Golgi morphology and Stx5 and Stx6 expression as well as acinar cell differentiation, whereas a putative holdase function, which should be retained in the Asna1(D74N) mutant (Voth et al., 2014), is unable to rescue these phenotypes. We further find that the CXXC(285-288) motif, which is predicted to affect the holdase function (Hemmingsson et al., 2010; Metz et al., 2006), also interferes with the ATPase-dependent Stx5 localization, presumably by affecting dimerization of Asna1/GET3 (Metz et al., 2006), thus precluding a direct test of the putative role for the holdase function of Asna1.

The redistribution of Stx5/Sed5 is a common hallmark phenotype observed in yeast GET3 mutants, *Asna1*-deficient pancreatic progenitor and β-cells, as well as in cardiomyocytes and hepatocytes in which the Asna1/TRC40 receptor WRB was disrupted, and thus indicates the functional loss of the ATPase-dependent GET3/Asna1/TRC40 pathway activity (Norlin et al., 2016; Powis et al., 2013; Rivera-Monroy et al., 2016; Schuldiner et al., 2008; Voth et al., 2014). The notion that membrane integration of Stx5, as well as that of other TA proteins, is directly mediated by the ATPase-dependent function of Asna1/GET3 relies heavily on studies in cell-free systems, whereas *in vivo* analysis show that many TA proteins are not dependent on Asna1/GET3 for their membrane insertion (Norlin et al., 2016; Rivera-Monroy et al., 2016; Schuldiner et al., 2008). However, the ability of Retro-2 to phenocopy the redistribution of Stx5 and Stx6 raises the possibility that these Golgi syntaxins are appropriately inserted into the ER membrane in *Asna1*-deficient cells but subsequently redistributed from the Golgi due to impaired vesicle transport. These two explanations are not mutually exclusive and additional experiments are required to fully resolve the role of Asna1 and retrograde transport for the localization of Stx5 and Stx6.

Regardless of the exact mechanism, several phenotypes of *Asna1*-deficient pancreatic progenitors and β-cells can be attributed to perturbed Retro-2-sensitive retrograde transport. First, as mentioned above, the redistribution of Stx5 and Stx6 can be reproduced by exposure of both islets (Norlin et al., 2016) and pancreatic explants to Retro-2. Second, impaired COPI-independent Golgi-to-ER retrograde transport and increased UPR signaling in *Asna1*-deficient β-cells is recapitulated by treatment with Retro-2 (Norlin et al., 2016). Finally, Golgi fragmentation, which is observed in progenitor cells but not in mature β-cells, is selectively reproduced by Retro-2 in progenitor cells but not in β-cells, further emphasizing the overlap between Retro-2-sensitive and *Asna1*-dependent processes, albeit in an apparent cell context-specific manner. In contrast, the induction of ISR signaling and the apoptosis observed in *Asna1^Panc−/−^* MPCs cannot easily be attributed exclusively to impaired Retro-2-sensitive retrograde transport. Like mature β-cells of *Asna1^ß−/−^* mice (Norlin et al., 2016), Sox9^+^ duct-like cells, Ngn3^+^ pro-endocrine cells and differentiated α-cells and β-cells present in the *Asna1^Panc−/−^* DPE remain refractory to apoptosis, despite apparent Stx5 and Stx6 redistribution as well as Golgi fragmentation. In addition, inhibition of retrograde transport by Retro-2 mimics the redistribution of Stx5 and Stx6, and the Golgi fragmentation in wild-type MPCs, but does not trigger extensive apoptosis or impair acinar cell differentiation. Thus, it appears that the requirement for *Asna1* ATPase function for progenitor cell survival is mediated by pathways that are independent of Stx5- and Retro-2-sensitive retrograde transport, but might mediate membrane insertion of other TA protein(s) that still remain to be defined. We cannot, however, exclude the possibility that poor solubility of Retro-2 precludes achieving a concentration that is sufficient complete inhibition of Asna1-dependent retrograde transport in pancreatic progenitor cells.

Several possibilities may account for the cell-specific differences in *Asna1*^−/−^ phenotypes. For example, the absence of Golgi fragmentation as observed in β-cells of *Asna1^ß−/−^* mice may be explained by the activation of UPR signaling that may help to maintain Golgi integrity (Norlin et al., 2016). Another possibility is that, dependent on the proliferative status of cells (i.e. pancreatic progenitor cells as compared with differentiated β-cells), the redistribution of Stx5 may prevent the re-assembly of the Golgi compartments after cell division (Rabouille et al., 1998). The proliferative status may also influence the apoptotic response. Cell types with limited proliferative potential, such as specified pro-endocrine cells and differentiated endocrine cells, appear refractory to apoptosis. In contrast, *Asna1^Panc−/−^* progenitor cell apoptosis, which may be partly p53 dependent, is observed among the proliferating cells in the MPC domain (tip cells) in both the dorsal and ventral pancreatic, as well as the proliferating duodenal, epithelia in *Asna1^Panc−/−^* mice. The link between p53, an oncogene that can induce cell cycle arrest and/or apoptosis in response to a variety of cellular stresses, and pancreatic progenitor cell apoptosis in *Asna1^Panc−/−^* mice, suggests that *Asna1* inactivation somehow interferes with cell cycle progression. Interestingly, cells deficient for one of the Asna1 ER-receptor subunits, CAML, exhibit mitotic defects that include chromosome mis-segregation (Liu et al., 2009). The causal link between *Asna1* deficiency and p53 activation, however, remains unknown and will require further analyses. To this end, it is worth noting that inactivation of RINT1, a component of the DSL1/NRZ and the RZZ complexes (Tagaya et al., 2014), interferes with retrograde transport and chromosome segregation, resulting in Golgi fragmentation, genomic instability and apoptosis (Grigaravicius et al., 2016), indicating a possible connection between the vesicle transport machinery and cell division control.

In summary, we show that *Asna1* in pancreatic progenitors has two functions: (1) ensuring retrograde transport pathway(s), thereby maintaining Golgi integrity; and (2) ensuring progenitor cell survival, possibly by preventing p53 activation. Both these functions rely, directly or indirectly, on the ATPase activity of Asna1, presumably by promoting insertion of a TA protein. We cannot, however, rule out putative ATPase-dependent functions of Asna1 that are unrelated to the actual membrane insertion of TA proteins, such as influencing protein degradation, or direct effects of Asna1 in regulating retrograde transport. Elucidation of the molecular mechanism(s) underlying the redistribution of Stx5 and Stx6 as well as the progenitor cell apoptosis observed in *Asna1^Panc−/−^* pancreatic and duodenal epithelium will require further analyses.

## MATERIALS AND METHODS

### Data reporting

For animal experiments, no sample-size estimate was calculated before the study was executed. The experiments were not randomized unless otherwise stated. Investigators were not blinded to allocation during experiments and outcome assessment. For *in vivo* data, each *n* value corresponds to a single mouse or mouse embryo. For *ex vivo* cultures, each *n* value corresponds to independent explants. If technical replicates were performed, then their mean was considered as *n*=1.

### Mouse strains and generation of *Asna1^Panc^* mice

*Ipf1-nlsCre* transgenic mice (Svensson et al., 2009) were bred with *Asna1^+/flox^* mice (Norlin et al., 2016), yielding *Asna1^+/flox^*, *Ipf1-nlsCre^+^* mice (denoted *Asna1^Panc+/−^*), which thus exhibit pancreas- and duodenum-specific Cre-mediated recombination from at least E10.5. Unless otherwise stated, male *Asna1^Panc+/−^* males were crossed with *Asna1^flox/flox^* females to yield *Asna1^Panc−/−^* mice. To achieve earlier inactivation of Asna1, we took advantage of the leakiness of the *Ipf1-nlsCre* transgene (Svensson et al., 2009) in the germ cell lineage of female *Asna1^Panc+/−^* mice, which thus transmit wild-type *Asna1* (*Asna1^+^*) or recombined *Asna1^null^* alleles. Hence, crossing male *Asna1^flox/flox^* mice with female *Asna1^+/flox^;Ipf1-nlsCre^+^* mice yields *Asna1^null/flox^;Ipf1-nlsCre^+^* mice (denoted *Asna1^null/Panc−^*). Cre-mediated recombination was visualized *in situ* by breeding a Rosa26^loxP-stop-loxP-LacZ^ transgene (Soriano, 1999) onto an *Ipf1-nlsCre^+^* or *Asna1^Panc+/−^* or *Asna1^Panc−/−^* background. For evaluation of autophagic degradation, *Asna1^Panc−/−^* mice were bred onto a background of GFP-LC3 transgenic mice (Mizushima et al., 2004) [provided by RIKEN BRC (RBRC00806)] and pancreas progenitor-specific deletion of Atg5 was performed by breeding Atg5^flox^ mice (Kuma et al., 2004) [provided by RIKEN BRC (RBRC02231)] with *Ipf1-nlsCre* mice. The genotypes of mice were determined by PCR analyses of genomic DNA samples extracted from tail biopsy specimens. For PCR primer sequences, see Table S3. PCR primers Asna-exon2-196F, ASNA_G_WT-A and Asna-G4R were used to detect the *Asna1^+^* (407 bp), *Asna1^flox^* (525 bp) and *Asna1^−^* (267 bp) alleles. PCR primers IPF1-5′3, IPF1-AR and CRE1 were used to detect the *Ipf1-nlsCre* transgene (950 bp) and endogenous *Ipf1* allele (700 bp). The GFP-LC3 transgenic construct and the endogenous LC3 allele were detected using PCR primers GFP(LC3) and LC3*rc3 (400 bp), and mLC3ex3GT and mLC3ex4AG (550 bp), respectively. The PCR primers ATG5exon3-1, ATG5check and ATG5short were used together to amplify the Atg5^flox^ (700 bp) and Atg5^WT^ (350 bp) alleles. Genetically modified mice were kept on a mixed background. Embryonic wild-type tissue for *in situ* hybridization and explants were obtained from crossing CBA males with B6 females. The animal studies were approved by the Institutional Animal Care and Use Committee of Umeå University and were conducted in accordance with the guidelines for the care and use of laboratory animals.

### Tissue isolation and preparation

Embryos were collected at selected stages. The day of the vaginal plug was considered embryonic day (E) 0.5. Embryonic tissue for immunohistochemical or *in situ* analysis were fixed in 4% PFA in PBS for 1 h, equilibrated in 30% sucrose in PBS, frozen and sectioned. For qPCR analysis or *ex vivo* culture experiments, the dorsal pancreatic buds were isolated using tungsten needles to remove as much of the surrounding mesenchyme as possible. Alternatively, as indicated in text, the mesenchyme was removed after proteolytic degradation with Dispase II (Roche).

### Immunohistochemistry and *in situ* hybridization

Tissue sections or intact cells were incubated for 20 min with blocking buffer [10% fetal bovine serum diluted in Tris-HCl (pH7.4), 0.15 M NaCl, 0.1%Triton-X100 (TBST)]. For mouse monoclonal primary antibodies, sections were additionally blocked with MOM reagent (30-60 min, diluted 1:30 in TBST) (Vector labs, MKB-2213). Tissues were incubated with primary antibodies (Table S1) overnight at 4°C followed by washes and incubation with fluorochrome-labeled secondary antibodies for 1 h. All antibodies were diluted in blocking buffer. TUNEL assay to detect apoptosis was performed according to manufacturer's recommendations (*in situ* cell death detection kit, Fluorescein, Roche, 11684795). *In situ* hybridization using digoxigenin-labeled probes was performed as described previously (Schaeren-Wiemers and Gerfin-Moser, 1993).

### Transmission electron microscopy (TEM)

Tissue samples were processed for TEM by Umeå Core Facility Electron Microscopy (UCEM). Briefly, tissue samples were fixed with 2.5% glutaraldehyde in 0.1 M sodium cacodylate buffer (pH7.4), post-fixed in 1% osmiumtetroxide, dehydrated and finally embedded in Spurr resin according to standard procedures. Sections were contrasted with uranyl acetate and lead citrate, and examined using a Jeol 1230 TEM. Micrographs were acquired using a Gatan MSC 600CW. The presence of electron-dense tight junctions between epithelial cells was used to distinguish them from mesenchymal cells.

### *Ex vivo* assays

Explants were cultivated on Millicell CM culture plate inserts (Merk, PICM01250) in explant medium: DMEM (Gibco, 21885) supplemented with 10% BCS, 10 mM HEPES (pH7.4) and 10 U/ml PEST (Gibco, 15140-122). Explant medium was further supplemented with vehicle (DMSO), Retro-2 (Calbiochem, 554715), pifithrin-α hydrobromide (PFT-α) (Tocris, 1267), Nutlin-3 (Tocris, #3984). To facilitate lentivirus infection, explants were split in half to expose the pancreatic epithelium and pre-incubated with concentrated virus particles for 2 h at 4°C.

### Lentivirus expression

Asna1 mutant cDNA was cloned after the EF1-promoter of the lentivirus expression vector EF.CMV.RFP (Addgene plasmid 17619, deposited by Linzhao Cheng), which also contains a separate CMV-driven RFP reporter to monitor transduction efficiency (Yu et al., 2003). The various expression vectors were co-transfected with envelope and packaging plasmids, pMD2.G and psPax2 (Addgene plasmids 12259 and 12260, deposited by Didier Trono), to produce virus particles as previously described (Szulc et al., 2006). Infection efficiency of pancreatic epithelia was similar for all constructs (∼1% of Ecad^+^ area) as monitored by the CMV-RFP expression. However, owing to imperfect correlation between expression from the EF1 and CMV promoters (Yu et al., 2003), the true efficiency of Asna1 re-expression is probably underestimated using this method.

### qRT PCR analyses

Individual dorsal pancreatic buds were used to prepare total RNA (RNeasy Micro Kit, Qiagen 74004) and cDNA (SuperScript III First-Strand Synthesis System, Life Technologies, 18080-051). Expression of the TBP was used to normalize expression levels. To ensure comparable Cq values, equal amounts (5 ng) of cDNA were used as template and Cq of the reference gene TBP was kept within ±1 cycle. Quantitative PCR (qPCR) analysis was performed using an ABI Prism 7000 sequence detection system and SYBR Green PCR Master Mix (ABI). Oligonucleotide sequences used for qPCR are listed in Table S2.

### Western blot analysis

Western blot expression data were normalized using α-tubulin expression. See supplementary Materials and Methods for further details.

### Image analysis and quantification

All comparisons were performed pairwise between *Asna1^Panc−/−^* and control tissue. For intensity comparisons and area quantification, images were captured using the same settings or modified to achieve similar background signal intensities. For quantification of immunopositive areas, the entire embryonic pancreas or pancreatic explant was sectioned (8 µm sections). Every 5th-15th section was collected for immunohistochemistry (5-12 sections/reaction) and all IHC sections were photographed and quantified using scripted procedures in ImageJ software, including software-based threshold algorithms to define immunopositive areas. Data from damaged sections were replaced by interpolation from adjacent sections.

### Statistical analyses

All numerical data are presented as mean±s.e.m. All statistical analyses were performed by heteroscedastic two-tailed Student's *t*-test. *P*<0.05 was considered statistically significant and denoted with: *P<0.05, **P<0.01, ***P<0.001.

## Supplementary Material

Supplementary information

Supplementary information
