# Supplementary material for: The ATPase activity of Asna1/TRC40 is required for pancreatic progenitor cell survival
Source: Development. 2018 Jan 1;145(1):dev154468. doi: 10.1242/dev.154468 (PMC5825870; doi:10.1242/dev.154468)
Supplement: Supplementary information [file develop-145-154468-s1.pdf]

SUPPLEMENTARY INFORMATION

Supplementary Methods

Western blot analysis

Tissue were collected in lysis buffer (2%SDS in 100mM Tris-HCl pH6.8), sonicated, and cleared by centrifugation. Protein expression was probed on western blots using antibodies (Table S1) diluted in blocking buffer (2.5% BSA in TBSTw [20mM Tris-HCl pH7.4, 0.15M NaCl, 0.1% Tween-20]) and quantified using chemiluminescent detection in ChemiDoc™ MP system (BioRad, #170-8280) followed by densitometry analyses of bands on Image Lab 4.1 using expression of α-tubulin to normalize expression levels.

Development • Supplementary information

Table S1. Primary antibodies

Antigen	Species	Supplier	Dilution
Amylase	Rabbit	Sigma-Aldrich #A8273	1:4000
Atf4	Rabbit	Cell Signaling #11815	1:100
cleaved Caspase-3	Rabbit	Cell Signaling #9661	1:1000
CPA	Rabbit	Serotec #1810-0006	1:2000
DBA lectin	NA	Vector laboratories #FL1031	1:100
EEA1	Mouse	Novus Biologicals #H00008411-M02	1:1000
ERGIC53	Rabbit	Sigma #E1031	1:200
E-cad	Rat	Zymed #13-1900	1:1000
Giantin	Rabbit	Abcam #ab24586	1:3000
Glucagon	Guinea pig	Linco #4031-01F	1:1000
Gm130	Mouse	BD Transduction Lab #610813	1:200
Insulin	Guinea pig	Dako #A0564	1:500
Ipf1	Rabbit	Produced in house (Ohlsson et al., 1993)	1:800
KDEL	Mouse	Abcam #ab12223	1:200
Lamp1	Rabbit	Novus Biologicals #NB120-19294	1:200
Ngn3	Rabbit	Produced in house (Selander and Edlund, 2002)	1:800
P53	Mouse	Abcam #ab187820	1:1000*
p62	Guinea pig	ProGen #GP62-C	1:800
Phospho-Histone H3	Rabbit	Upstate # 06-570	1:1000
Ptf1a	Rabbit	Produced in house (Li and Edlund, 2001)	1:800
Sec22b	Rabbit	Osenses #OSS00040W	1:200
Sec61β	Rabbit	Proteintech #15087-1-AP	1:100
Sox9	Rabbit	Millipore # ab5535	1:1000
Stx5	Rabbit	Synaptic System #110 053.	1:400<br>1:5000*
Stx6	Mouse	BD Transduction Lab #610635	1:100
TGN46	Rabbit	Abcam #ab16059	1:200
α-tubulin	Mouse	Sigma-Aldrich #T5168	1:10000*

* dilution for western blot

Development • Supplementary information

Table S2. Primers used in qRT-PCR analyses

Gene	Forward (5'-3')	Reverse (5'-3')
Asna1 (exon2)	TTCCACAGACCCAGCTCACAA	GCCTTTGACCTTGGTAGGCAC
Atf3	GTGTCGAAACAAGAAAAAGGAGAAG	TCTCTGACTCTTTCTGCAGGCAC
Atf4	GGAATGGCCGGCTATGG	TCCCGGAAAAGGCATCCT
BiP	TCAGCCAATTATCAGCAAACTCT	TTTTCTGATGTATCCTCTTCACCAGT
Chop10	CAGAAGGAAGTGCATCTTCATAC	GACTGCACGTGGACCAGGTT
Dnajc3	GACAGCTAGCCGACGCCTTA	GTCACCATCAACTGCAGCGT
Herpud1	CATGTACCTGCACCACGTCG	GAGGACCACCATCATCCGG
HO-1	CTAGCCCCAGTCCGGTGATG	CAAATCCTGGGGCATGCTG
Hsp90b1	TGTATGTACGCCGCGTATTCA	TCGGAATCCACAACACCTTTG
Nrf2	TTTCAGCGTGGCTGGGGATA	TGACCATGAGTCGCTTGCCC
Nqo1	CGCCTGAGCCCAGATATTGT	CTGCAATGGGAACTGAAATATCAC
Pdia4	TGACCCGGCCTACTTGCA	GTGTGGTGAAACTTGTAATCTTCTCTCA
Tbp	GAATTGTACCGCAGCTTCAAAA	AGTGCAATGGTCTTTAGGTCAAGTT
Trib3	ACTGTGAGAGGACGAAGCTGG	TCACGCAGGCATCTTCCA
XBP1-spliced	CTGAGTCCGCAGCAGGTGCAG	GTCCATGGGAAGATGTTCTGG

Development • Supplementary information

Table S3. Primers used for PCR genotyping.

Genotyping primers (5'-3')	
Asna-exon2-196F	TGTCGAAGGGACGTGAGAGTG
Asna-G4R	TGGTGGCTGCTGGTAGGTA
ASNA_G_WT-A	GGGCAAAGCGAACTGA
IPF1-5´3	GGGAAGAGGAGATGTAGACTT
IPF1-AR	GAGCTGAGCTGGAAGGT
CRE1	GTGCAAAACAGGCTCTAG
GFP(LC3)	TCCTGCTGGAGTTCGTGACCG
LC3*rc3	TTGCGAATTCTCAGCCGTCTTCATCTCTCTCGC
mLC3ex3GT	TGAGCGAGCTCATCAAGATAATCAGGT
mLC3ex4AG	GTTAGCATTGAGCTGCAAGCGCCGTCT
ATG5exon3-1	GAATATGAAGGCACACCCCTGAAATG
ATG5check	ACAACGTCGAGCACAGCTGCGCAAGG
ATG5short	GTACTGCATAATGGTTTAACTCTTGC

Development • Supplementary information

Figure S1 (related to Figure 1)

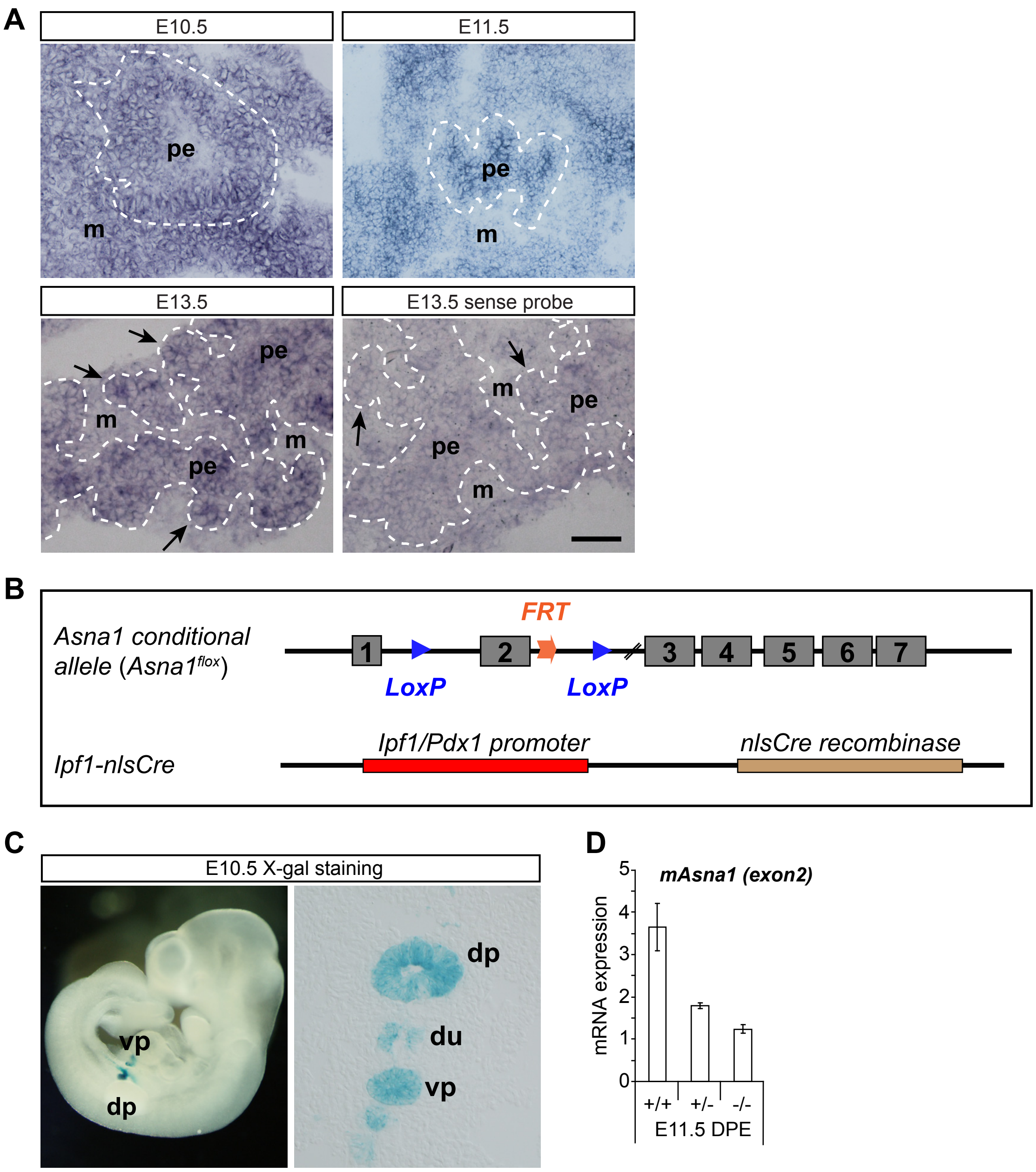

Fig. S1.

(A) *In situ* hybridization using *Asna1* antisense and sense probes showing expression of *Asna1* in the pancreatic epithelium of E10.5-E13.5 wildtype embryos.

(B) Schematic presentation of the conditional Asna1 allele (*Asna1^{flox}*) and the Ipf1-nlsCre transgenic construct used to generate *Asna1$^{Panc-/-}$* mice.

(C) X-gal staining of E10.5 *Ipf1-nlsCre*;*Rosa26$^{loxP\text{-}stop\text{-}loxP\text{-}LacZ+/-}$* (Soriano, 1999) embryos showing *Ipf1/CRE* mediated reporter, i.e. LacZ, expression in pancreatic and duodenal progenitor epithelia.

(D) qRT-PCR analysis of *Asna1(exon2)* expression in dorsal pancreatic epithelium stripped of mesenchyme (DPE) isolated from E11.5 *Asna1$^{Panc+/+}$*, *Asna1$^{Panc+/-}$* and *Asna1$^{Panc-/-}$* mice (n=4).

Scale bar in (A), 50µm. Dashed lines in (A) delimit pancreatic epithelia. Arrows in (A) indicate pro-acinar tip cells. dp, dorsal pancreas; du, duodenum; m, mesenchyme; pe, pancreatic epithelium; vp, ventral pancreas.

Development • Supplementary information

Figure S2 (related to Figure 1)

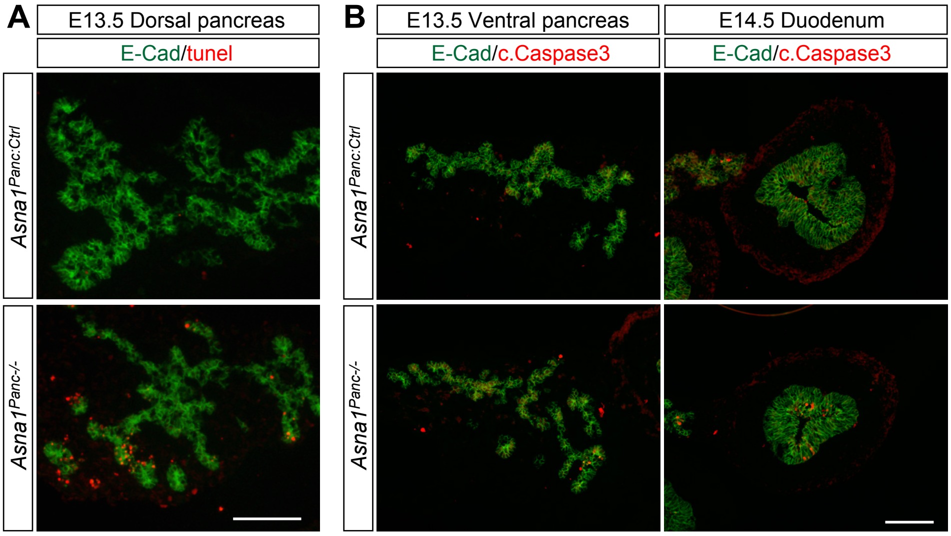

Fig. S2.

(A) Combined tunel staining and immunohistochemistry of dorsal pancreatic sections from E13.5 *Asna1*$^{Panc:Ctrl}$ and *Asna1*$^{Panc-/-}$ embryos (n=2) using tunel assay to detect DNA fragmentation (red) in apoptotic cells combined with antibodies against E-cadherin (E-cad; green) to detect pancreatic epithelium.

(B) Immunohistochemistry of E13.5 ventral pancreas and E14.5 duodenum from *Asna1*$^{Panc:Ctrl}$ and *Asna1*$^{Panc-/-}$ embryos (n=3) using antibodies against E-cadherin (E-cad; green) and cleaved Caspase3 (c.Caspase3; red).

Scalebar, 100µm in (A and B)

Figure S3 (related to Figure 2)

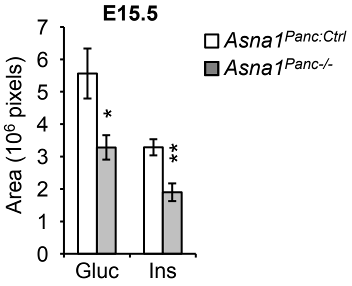

Fig. S3.
Quantification of total glucagon^{+} (Gluc) and insulin^{+} (Ins) area in the DPE from *Asna1*$^{Panc:Ctrl}$ and *Asna1*$^{Panc-/-}$ embryos at E15.5 (n=5 respectively).
Data are presented as mean±SEM, *p<0.05, **p<0.01, (student *t* test).

Development • Supplementary information

Figure S4 (related to Figure 3)

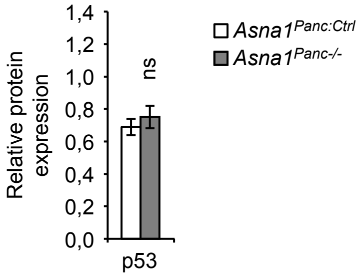

Fig. S4.
Western blot quantification of p53 protein levels relative to α-tubulin in E13.5 dorsal pancreas buds from *Asna1*$^{Panc:Ctrl}$ (n=6) and *Asna1*$^{Panc-/-}$ embryos (n=3). Data are presented as mean±SEM, ns=not significant (student t test).

Development • Supplementary information

Figure S5 (related to Figure 4)

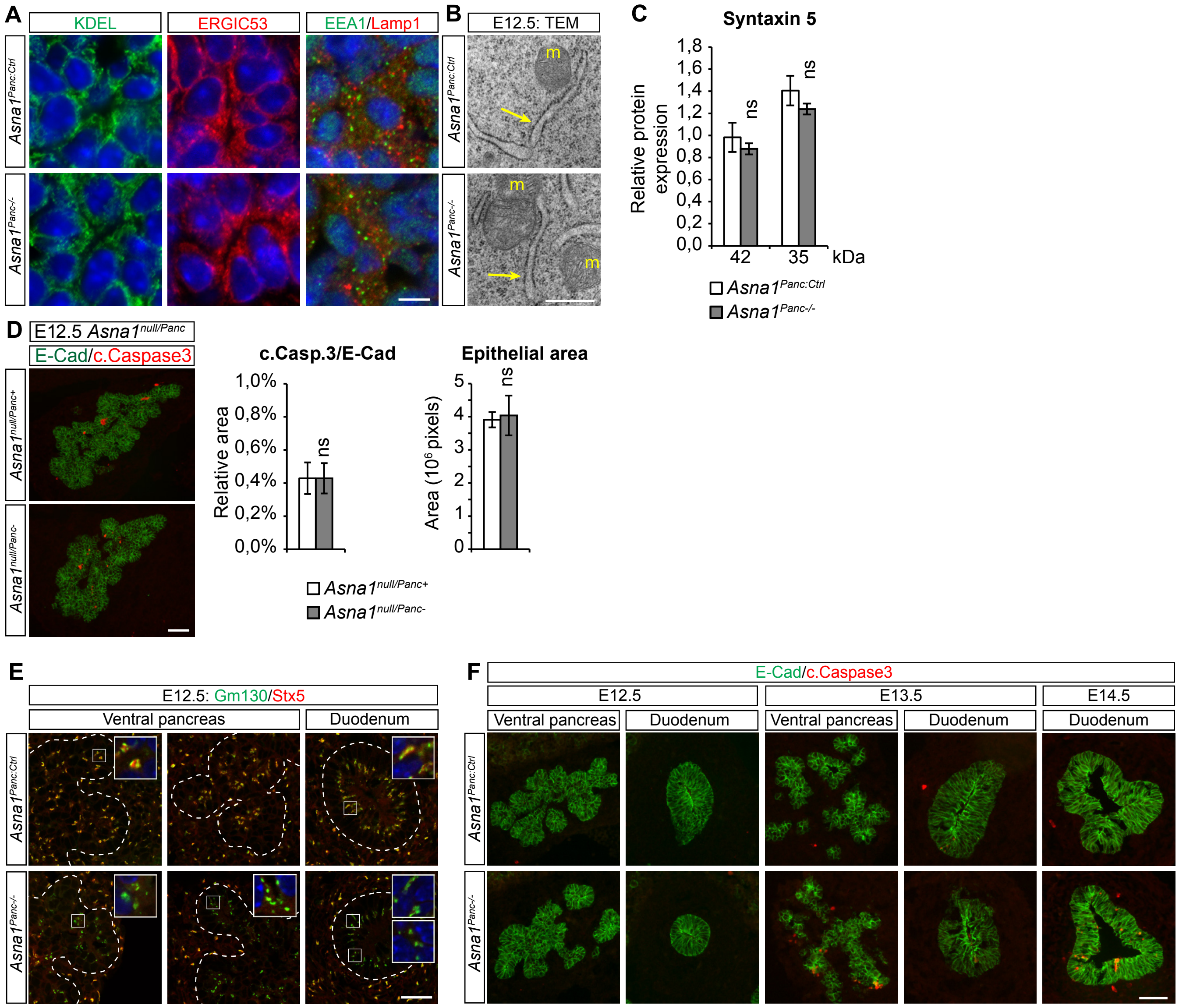

Fig. S5.

(A) Immunohistochemistry of dorsal pancreatic sections from E12.5 *Asna1*$^{Panc:Ctrl}$ and *Asna1*$^{Panc-/-}$ embryos (n=3) using antibodies against KDEL (green), ERGIC (red), EEA1 (green) and Lamp1 (red).

(B) Transmission electron micrograph (TEM) of dorsal pancreatic sections from E12.5 *Asna1*$^{Panc:Ctrl}$ and *Asna1*$^{Panc-/-}$ embryos (n=3) showing mitochondria (m) and rough ER (yellow arrows).

(C) Western blot quantification of the 35kDa and 42kDa isoforms of Stx5 protein levels relative α-tubulin in E12.5 dorsal pancreas buds from *Asna1*$^{Panc:Ctrl}$ (n=3) and *Asna1*$^{Panc-/-}$ embryos (n=3).

(D) Representative immunohistochemistry and quantification of dorsal pancreas from E12.5 *Asna1*$^{null/Panc+}$ and *Asna1*$^{null/Panc-}$ (n=3) using antibodies against E-cadherin (E-cad; green) and cleaved Caspase 3 (c.Caspase3; red).

(E) Immunohistochemistry of ventral pancreas and duodenum from E12.5 *Asna1*$^{Panc:Ctrl}$ and *Asna1*$^{Panc-/-}$ embryos (n=3) using antibodies against Gm130 (green) and Stx5 (red). Insets show magnification of selected areas (squares). Stx5 expression is reduced in the Golgi of E12.5 *Asna1*$^{Panc-/-}$ ventral pancreas and duodenum. To illustrate different degrees of Golgi fragmentation (Gm130 in green) at this stage, the ventral pancreas from two different animals are shown and two regions of the *Asna1*$^{Panc-/-}$ duodenal epithelium are magnified (insets).

(F) Immunohistochemistry of ventral pancreas and duodenum from E12.5, E13.5 and E14.5 *Asna1*$^{Panc:Ctrl}$ and *Asna1*$^{Panc-/-}$ embryos (n=3) using antibodies against E-cadherin (E-cad; green) and cleaved Caspase 3 (c.Caspase3; red). Increased number of c.Caspase3^{+} apoptotic cells are observed at E13.5 in the ventral pancreas and at E14.5 in the duodenum, which is 1-2 days after the first signs of perturbed Golgi morphology and Stx5 expression (compare with panel [E]).

DAPI (blue) indicate nuclei in (A). Dashed lines in (E) delimit pancreatic or duodenal epithelia. Scalebars: 10µm in (A), 0.5µm in (B), 50µm in (D and F), and 25µm in (E).
